# Vaccine development for mosquito-borne viral diseases

**DOI:** 10.3389/fimmu.2023.1161149

**Published:** 2023-05-12

**Authors:** Zhiwei Huang, Yuxuan Zhang, Hongyu Li, Jiajie Zhu, Wanchen Song, Keda Chen, Yanjun Zhang, Yongliang Lou

**Affiliations:** ^1^ School of Laboratory Medicine and Life Sciences, Wenzhou Medical University, Wenzhou, China; ^2^ Shulan International Medical College, Zhejiang Shuren University, Hangzhou, China; ^3^ School of Medical Technology and Information Engineering, Zhejiang Chinese Medical University, Hangzhou, China; ^4^ Department of Microbiology, Zhejiang Provincial Center for Disease Control and Prevention, Hangzhou, China

**Keywords:** vaccine, Zika virus, Chikungunya virus, dengue virus, mosquito-borne viral disease

## Abstract

Mosquito-borne viral diseases are a group of viral illnesses that are predominantly transmitted by mosquitoes, including viruses from the Togaviridae and Flaviviridae families. In recent years, outbreaks caused by Dengue and Zika viruses from the Flaviviridae family, and Chikungunya virus from the Togaviridae family, have raised significant concerns for public health. However, there are currently no safe and effective vaccines available for these viruses, except for CYD-TDV, which has been licensed for Dengue virus. Efforts to control the transmission of COVID-19, such as home quarantine and travel restrictions, have somewhat limited the spread of mosquito-borne viral diseases. Several vaccine platforms, including inactivated vaccines, viral-vector vaccines, live attenuated vaccines, protein vaccines, and nucleic acid vaccines, are being developed to combat these viruses. This review analyzes the various vaccine platforms against Dengue, Zika, and Chikungunya viruses and provides valuable insights for responding to potential outbreaks.

## 1 Introduction

Mosquito-borne viral diseases are a significant public health burden worldwide. With increased international travel, the speed and range of these diseases are expanding, resulting in higher chances of virus mutation. The Flaviviridae family comprises several viruses, including Dengue virus (DENV), Zika virus (ZIKV), West Nile virus (WNV), yellow fever virus (YFV), and Japanese encephalitis virus (JEV). These viruses have caused outbreaks in many regions, and the number of cases has been increasing in recent years. Despite posing a significant threat to human health during the initial stages of the outbreak, the spread of YFV and JEV was mostly contained after several safe and effective vaccines became available (1). However, The range and prevalence of DENV and ZIKV are increasing year by year. Shepard et al. pointed out that approximately 400 million dengue cases and 22,000 deaths occur worldwide annually and are categorized as a “neglected tropical disease” (2, 3). Based on epidemiological updates in 2022, 2,735,694 cases of arboviral diseases were reported in the Americas, which contained 2463188 (90%) cases of dengue, 242,924 cases of chikungunya, and 29,582 cases of ZIKV (4). The World Health Organization released an announcement in February 2016 declaring ZIKV a critical public health emergency, which deserved international attention. In November 2016, WHO again stated that the virus was an ongoing challenge requiring intense action, not just a public health emergency of international concern. ZIKV remains a serious challenge for mankind (5).

Since the first local Chikungunya virus (CHIKV) transmission occurred in Caribbean countries and territories, CHIKV subsequently spread rapidly throughout the Americas (6). Subsequently, CHIKV spread across the Asian and European continents, resulting in several epidemics. In 2004, outbreaks in India, Italy, and France led to a cumulative total of 1.3 million reported cases of CHIKV. Compared to other viruses in the Togaviridae family, the epidemiological impact of CHIKV and the progress of vaccine research warrant greater attention (7). Therefore, scientists have promoted and facilitated vaccine research and evaluation with the aim of halting three Mosquito-borne viral diseases transmission.

**Figure 1:**
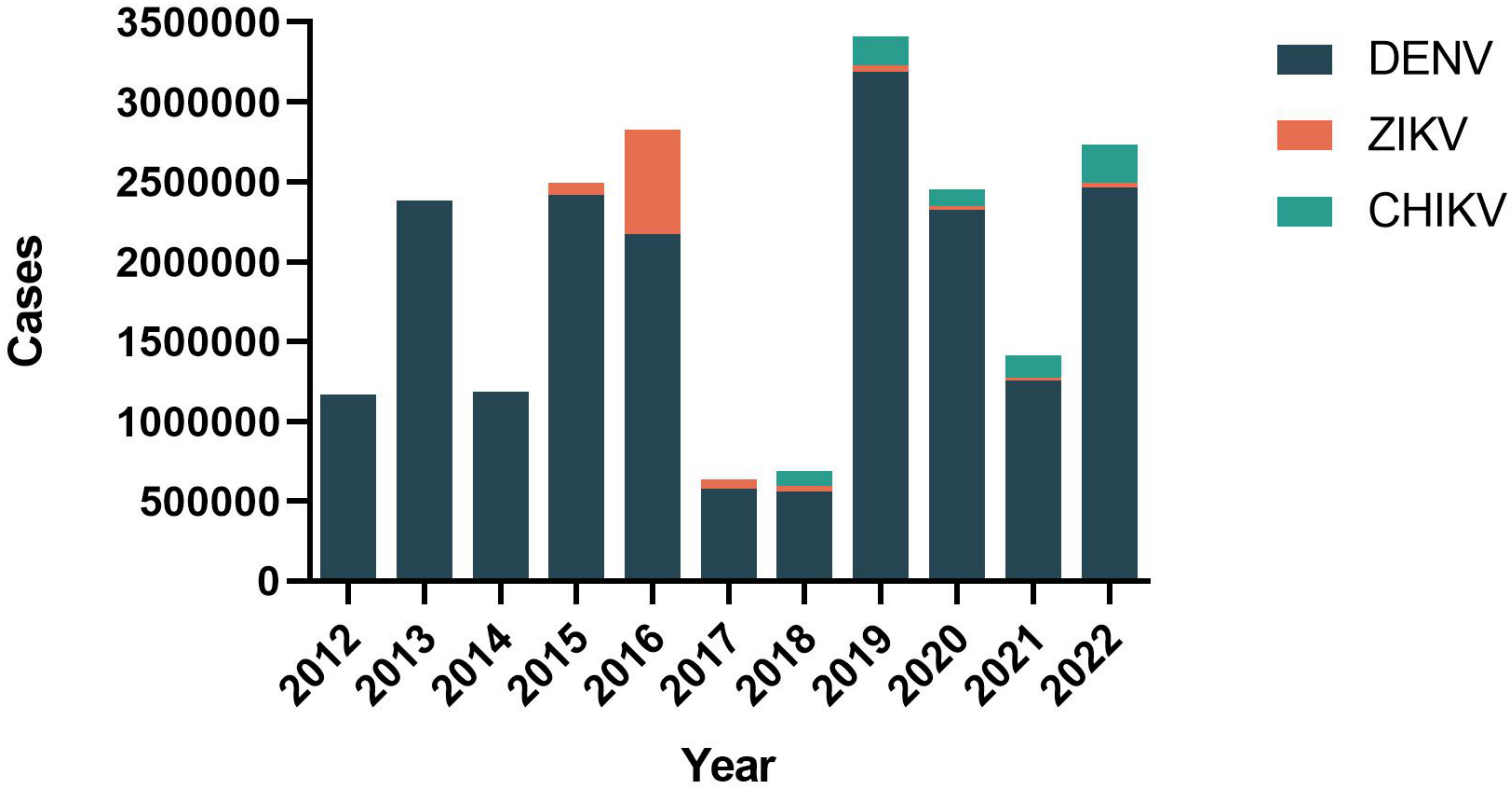
Distribution of reported cases of dengue, chikungunya, and Zika by year of report. Region of the Americas, 2012–2022.

**Figure 2:**
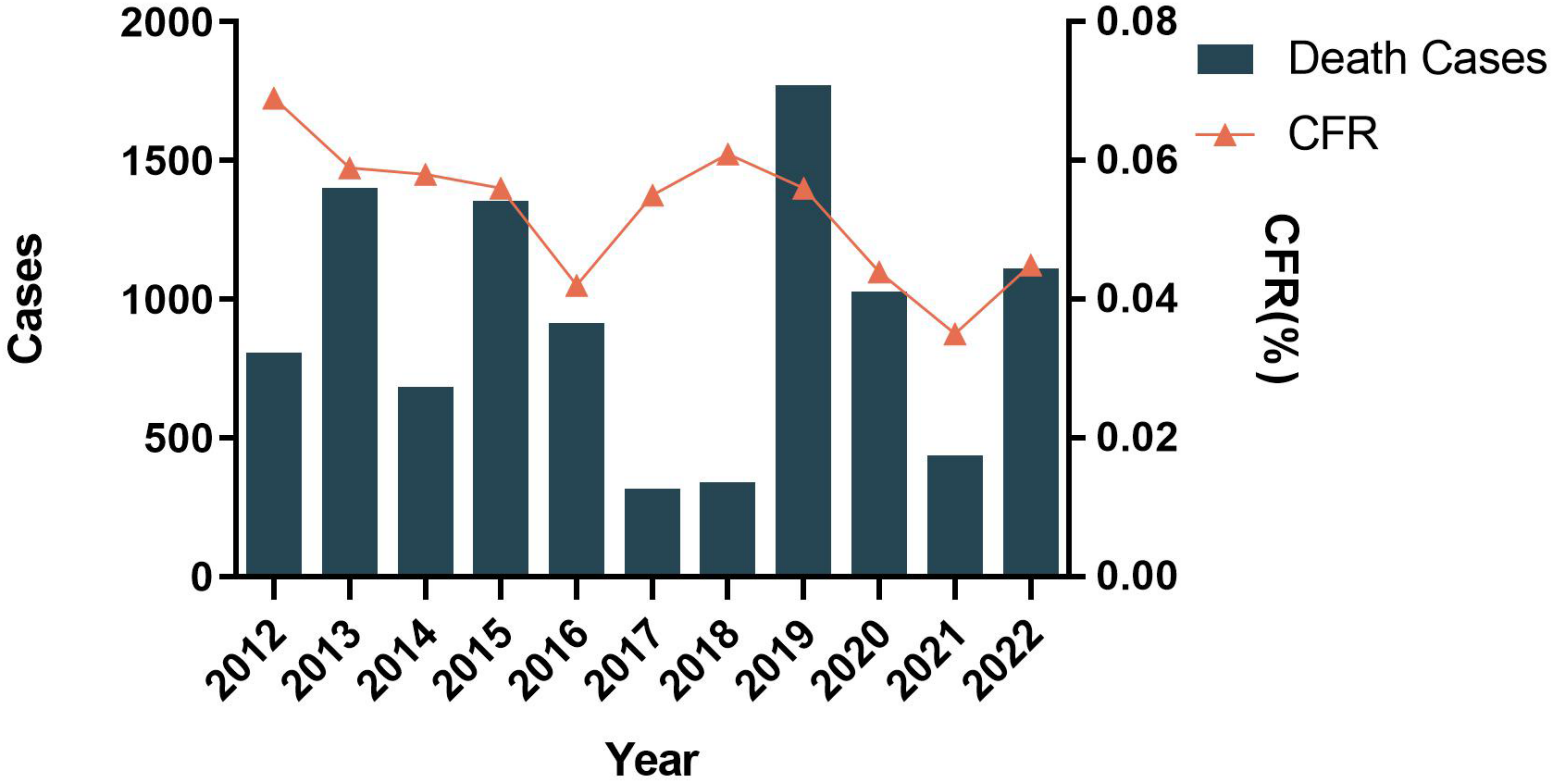
Distribution of reported deaths and CFR of dengue by year of report. Region of the Americas, 2012–2022.

Based on the data pooled by the Pan-American Health Organization (PAHO) (4), we have summarized the distribution of reported cases of dengue, chikungunya, and Zika according to the year of the report, from 2012 to 2022 (**Figure 1**). Although the reported cases of dengue virus (DENV) decreased historically in 2017 and 2018 (579027 in 2017, 561689 in 2018), the case fatality rate (CFR) was maintained at 0.05% (**Figure 2**). Besides, the co-transmission of other arboviruses, such as CHIKV and ZIKV, was reported in the Americas. Despite Dengue cases decreasing in the most recent 3 years in the Americas (3,190,778 in 2019, 2,326,115 in 2020, 1,254,648 in 2021), which is largely due to the improvement of insect vector control, medical treatment, and the impact of coronavirus disease 2019 (COVID-19), there is still an urgent need for effective vaccines to eradicate dengue.

These three viral diseases are global public health crisises, especially in endemic countries with limited medical support. The historical record proves that vaccines are the only effective way to interrupt the spread of infectious diseases. Furthermore, when compared with cutting off the dissemination route or controlling the source of infection, vaccination has the best record for reducing morbidity and mortality. However, among Togaviridae and Flaviviridae families, the severe epidemiological distribution situation and the severe immunopathological phenomena greatly increase the difficulty of vaccine development for these three viruses. This review aims to: (a) Describe in detail the development of vaccines to combat DENV, ZIKV, and CHIKV; (b) analyze the protection efficacy and the titers of neutralizing antibodies produced by different vaccines targeting these three Mosquito-borne viral diseases; (c) provide a theoretical basis for vaccine research and suggest possible vaccine strategies to deal with future epidemics of Mosquito-borne viral diseases.

## 2 Candidate targets for vaccines

DENV, ZIKV, WNV, YFV, and JEV belong to the Flavivirus genus within the Flaviviridae family. Flaviviruses are enveloped, positive-sense, single-stranded RNA viruses. All of them encode three structural proteins [capsid (C), premembrane (P), and envelope (E)] and seven nonstructural proteins (NS1, NS2A, NS2B, NS3, NS4A, NS4B, and NS5) (8). The E protein is the main immunogen for host protective immunity and induces neutralizing antibodies (9). The E protein consists of three domains: Domain I participates in the structural rearrangements of the E protein required for fusion, and acts as a hinge link between domain II and III (10). Domain II contains the fusion loop that facilitates pH-dependent membrane fusion between the virus and its host. Domain III is the receptor binding region, which serves as putative binding domain for E protein (11). The premembrane protein might assist the folding and assembly of the E protein. In addition to forming nucleocapsids, the capsid protein is responsible for packaging the viral genome (12). The non-structural proteins play a crucial role in virus replication. Many researchers have chosen NS1 as a diagnostic target because it is the only protein secreted by infected host cells. NS1 also participates in the replication and assembly of the virus (13). NS3 and NS5 are mainly involved in CD8^+^ T cell activities (14). NS3 activates proproteinase-dependent and proproteinase-independent pathways that inhibit type I interferon production in conjunction with NS2B and NS4A to inhibit the interferon pathway. NS5 is an RNA-dependent RNA polymerase (RdRp) used for genome replication, acts as a methyltransferase for 5′ RNA cap formation and methylation, and is a type I interferon antagonis (11). There are four different serotypes of DENV; therefore, the different structures and formulations in the available vaccines result in different antibody efficacies against the different serotype target antigens.

CHIKV is an enveloped virus with a positive sense, single-stranded RNA genome. The genome of CHIKV encodes the non-structural proteins (nsP1–4) and the structural proteins (capsid, small 6K protein, and envelope proteins E1, E2, and E3). The icosahedral shell formed by E1 and E2 at the surface of the virion enhances the invasion of susceptible cells (15). The nsP1 protein is used for capping and methylating the viral genome. The nsP2 protein acts as a cofactor of the viral polymerase complex (16). As an RdRP, nsP4 promotes the formation of genomic and sub-genomic viral RNAs (17). The function of nsP3 is clear.

## 3 Vaccine candidates

Researchers have applied several strategies in vaccine research, including live attenuated vaccines, inactivated vaccines, DNA vaccines, mRNA vaccines, protein vaccines, and viral vector vaccines. **Supplementary Table 1** summarizes these vaccine candidates against these Mosquito-borne viral diseases.

### 3.1 Dengue vaccine candidates

**Figure 3:**
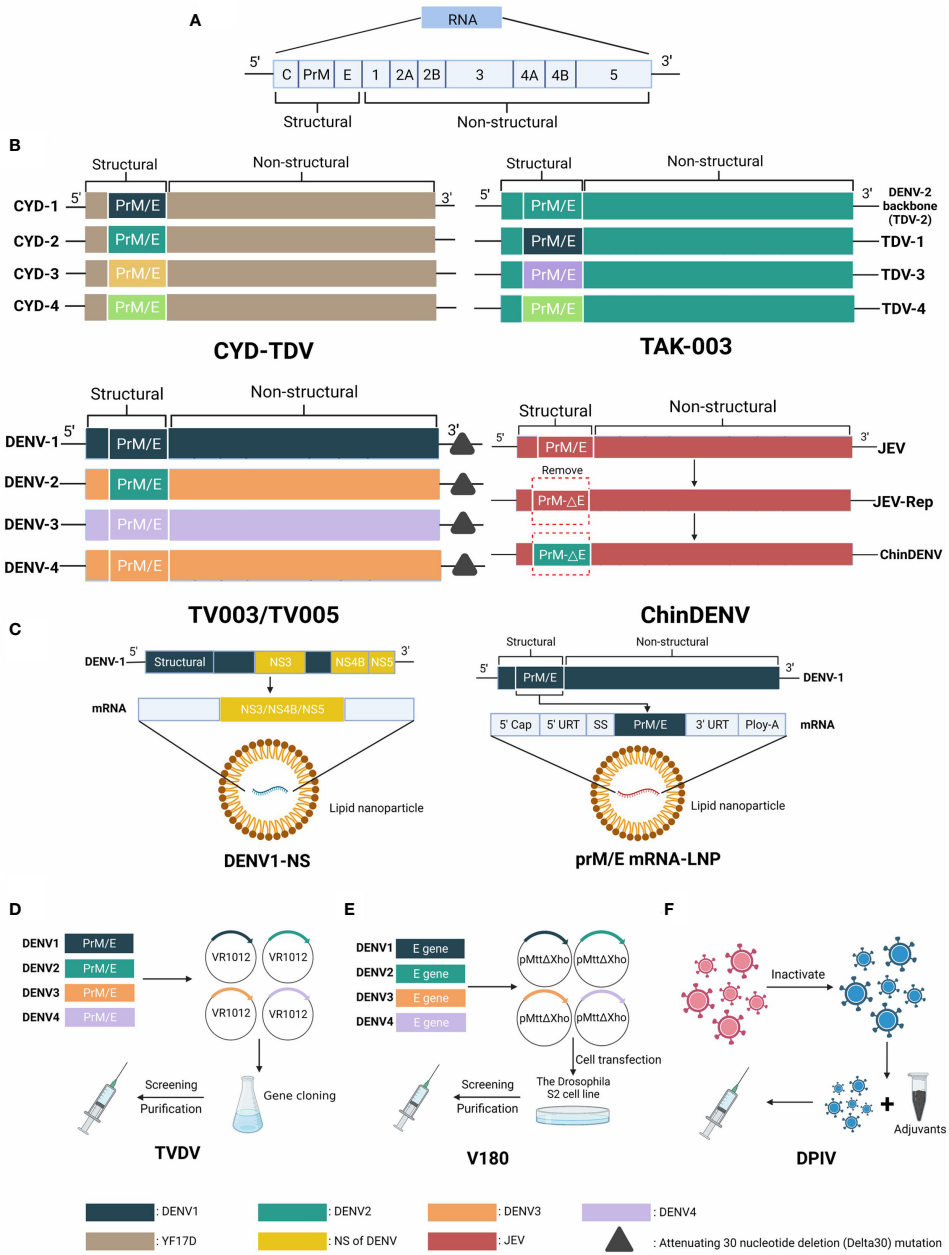
The genome of Dengue virus and structural schematic diagrams of DENV vaccine candidates. **(A)** The genome is approximately 11,000 kb in length and is an enveloped, positive-sense, single-stranded RNA virus, encoding three structural and seven nonstructural proteins. **(B)** Four structure diagrams of live attenuated vaccine candidates, distinguished by different colors. CYD-TDV takes YFV 17D as the backbone and replaces prM/E genes with four serotypes. The backbone of TAK-003 is a weakened DENV2 strain (PDK-53) that contains the prM/E parts of all serotypes. TV003/TV005 has deletions in the 3´ untranslated region. ChinDENV applies JEV vaccine strain SA14-14-2 as the backbone and substitutes the prM/E gene of DENV. **(C)** Two types of mRNA vaccine candidates using different strategies. DENV1-NS contains the NS3, NS4B, and NS5 genes. prM/E mRNA-LNP encodes the prM/E structural proteins. **(D)** The plasmids VR1012 of TVDV (DNA vaccine) contain prM/E genes of four serotypes of DENV. **(E)** Protein vaccine V180 consists of a truncated protein DEN-80E expressed by the Drosophila S2 cell line. **(F)** Inactivated vaccine DPIV contains four serotypes of attenuated DENV in primary dog kidney (PDK) cells.

Dengue, one of the most widespread Mosquito-borne diseases in the world, is transmitted by Aedes mosquitoes. Dengue infections are very expensive for families and society as a whole (18). Genetically and antigenically, dengue virus can be categorized into four types (DENV-1, DENV-2, DENV-3, DENV-4). They belong to the Flavivirus genus, family Flaviviridae (19). Dengue virus infection has a variety of clinical manifestations in addition to dengue hemorrhagic fever (DHF) and dengue shock syndrome (20). The C, M, E, NS1, NS2A, NS2B, NS3, NS4A, and NS5 proteins assist DENV to invade the host by hijacking host cell machinery and escaping from the host immune responses (21). A structural diagram of current vaccines DENV is shown in **Figure 3**.

#### 3.1.1 Live attenuated vaccine candidates

Live attenuated vaccines, which consist of a living pathogen that is less virulent or avirulent, can provide long-term immune protectivity and protective antigens (22). There is currently only one licensed tetravalent live attenuated vaccine, CYD-TDV (Dengvaxia). In CYD-TDV, the corresponding genes in yellow fever virus (YFV) 17D were replaced with the genes encoding PrM/E from the four serotypes of DENV (23). The YFV 17D vaccine is also manufactured by Sanofi Pasteur as a chimeric vaccine for dengue and yellow fever viruses. The viscus injury and neurovirulence of CYD-TDV are weaker than the YFV 17D vaccine as a backbone and CYD-TDV has no risk of spreading to mosquitoes (23, 24). A phase IIb clinical trial in Thailand, which involved 4002 children aged 4 to 11 injected with three doses at 0, 6, and 12 months, revealed that the protective effectiveness of the vaccine was 30.2% in general. In detail, the protectivity against the four serotypes after one injection was different (61.2% against DENV-1, 3.5% against DENV-2, 81.9% against DENV-3, and 90.0% against DENV-4). The geometric mean titers (GMTs) increased after each injection. Within four weeks of the third injection, the GMTs peaked in the range of 146 against DENV1 and 405 against DENV3. At 1 year after the third injection, GMTs decreased in the range of 76.5 to 153 higher than baseline (25). These data suggested that there are conspicuous differences between the overall protectivity and GMTs *in vitro*. Thus, CYD-TDV can be considered a safe dengue vaccine and its overall efficacy depends largely on age, serostatus, and DENV serotype (26).

Takeda developed TAK-003 to replace the attenuated DENV-2 (named PDK-53) with the corresponding premembrane and envelope gene regions from DENV-1, DENV-3, and DENV-4. Compared with CYD-TDV, TAK-003 can not only stimulate host immune humoral immunity against DENV, but also induce cellular immunity because the DENV-2 backbone consists of non-structure gene sequences (27). A phase I trial demonstrated that TAK-003 is immunogenic, with an acceptable reactogenicity (28). In a phase II trial carried out in Singapore, using the multicolor FluoroSpot (MCF) assay, TAK-003 produced both type-specific memory B cells (MBCs) and cross-reactive MBCs in all macaques and clinical trial participants. Moreover, the proportion of type-specific MBCs was higher than cross-reactive MBCs (29). TAK-003 achieved neutralizing antibody titers of 144–1730 and 53–656 in participants who were originally serologically negative at 30 and 360 days after the two doses, respectively (30). Vaccination with TAK-003 resulted in a DENV-specific MBC response, as indicated by the study results. Another phase II trial demonstrated that the GMTs after one dose were lower than the GMTs after a two-dose primary series or one primary dose plus a one-year booster (31). In general, compared with the CYD-TDV vaccine, TAK-003 appeared less serostatus dependent and had better prospects.

The National Institute of Allergy and Infectious Diseases (NIAID) has reported a dengue live attenuated tetravalent vaccine (LATV). The core principles of LATV comprise two strategies: Deletions in the 3´ untranslated region and structural gene chimerization (32). It consists of four components: DENVs attenuated by at least one deletion in the 3’ untranslated region of three full-length forms and a chimeric virus in which DENV-4 in the DENV-4D30 background was superseded by the premembrane and envelope proteins of DENV-2 (33). Based on different vaccine formulas, two vaccines were produced: TV003 and TV005. Similar to TAK-003, TV003/TV005 contains DENV-1, DENV-3, and DENV-4 as backbones; therefore, it can elicit a host cellular immune response. Aka TV003 produced neutralizing antibody titers of 93.96–445.39 in participants after one dose (34). Compared with one dose and two doses after one year, the results showed that the mean peak titer of neutralizing antibodies declined from one dose (DENV-1: 80, DENV-2: 107, DENV-3: 178, DENV-4: 255) to two doses after one year (DENV-1: 31, DENV-2: 64, DENV-3: 65, DENV-4: 73). This trial demonstrated that the neutralizing antibody level after a single dose might be sufficient to resist the virus (35).

There has been widespread use of a licensed live vaccine against Japanese encephalitis virus (named strain SA14-14-2) in China and other Asian countries. In addition to its inherent robustness, and remarkable potency and efficacy, SA14-14-2 also has a good safety profile. Therefore, adopting SA14-14-2 as the genetic base, chimeric dengue vaccines (ChinDENVs) were developed containing the premembrane and envelope gene regions of DENV (36). Their safety profiles and immunogenicity were proven in a pre-clinical trial (37).

Among these live attenuated vaccines, only the above three have entered clinical trials, namely CYD-TDV, TAK-003 and TV003/TV005. It is important to note that CYD TDV showed the highest protection against DENV3 and DENV4, and the lowest protection against DENV2. In contrast, TAK-003 showed the highest protective efficacy toward DENV2 and the lowest protective efficacy toward DENV4. By contrast, Aka TV003 exhibited relatively balanced protection efficacy. It has been reported that the cellular immune responses mainly involve the recognition of NS1, NS2A, and NS3 (38). TAK 003 was built around DENV2, while CYD-TDV was built around YFV; therefore, TAK-003 contained the capsid protein, all seven nonstructural proteins, and all seven structural proteins; however, CYD-TDV did not. This probably explains why TAK-003 showed the highest protective efficacy against DENV2. CYD-TDV might induce only responses from CD8+ T cells specific for NS3 in YF-17D and CD4+ T cells specific for DENV serotypes (39). There was a strong T cell response against the TV003 vaccine candidate because it contained the nonstructural protein of three other DENV serotypes. Therefore, TV003 produced good antibody titers against different serotypes of DENV after single-dose vaccination.

#### 3.1.2 Inactivated vaccine candidates

As a result of a collaboration between the Walter Reed Army Institute of Research (WRAIR), GlaxoSmithKline (GSK) Vaccines, and Fiocruz, a candidate tetravalent purified inactivated vaccine (TPIV) is currently under development. A DPIV comprises four strains of a live virus that have been attenuated by serial culture in primary dog kidney cells (40). The WRAIR carried out a phase I human trial in a limited subgroup of healthy, flavivirus uninfected volunteers, which focused on comparing four different DPIV formulations with aluminum hydroxide (alum) adjuvant or GSK’s adjuvant systems AS01E and AS03B for safety and immunogenicity (41). DPIV produced neutralizing antibody titers that varied considerably between adjuvants. At 28 days after the second dose, the group involving 4 μg of alum adjuvant produced neutralizing antibody titers of 141–191 compared to the group involving 1 μg of alum adjuvant, which produced neutralizing antibody titers of only 20–55. However, one year after the second vaccination, the antibody titers measured in the two groups of participants were not very different. In contrast, the other two adjuvants (AS01_E_ and AS03_B_) produced much stronger neutralizing antibody titers. Even after one year, the antibody titers produced by the AS01_E_ and AS03_B_ groups were nearly twice as high as those of the alum group (41). At one month after the second exposure, the alum-adjuvanted formulations, AS03B-adjuvanted formulations, and AS01E-adjuvanted formulations all produced antibody responses. Moreover, further analysis revealed that DPIV adjuvanted with AS03B produced superior antibody responses that continued to be detectable three years after the second dose (42).

#### 3.1.3 mRNA vaccine candidates

With the development *in vitro*-synthesized mRNA technology, the stability and safety of mRNA vaccines have been enhanced. After injection of the mRNA encoding virus proteins, the virus proteins will be synthesized *in vivo*, prompting a host immune response to the virus (43). Lipid nanoparticles (LNPs), as a type of self-adjuvant, can induce an innate immune response (44). Therefore, the *in vitro*-synthesized mRNA is usually encapsulated in LNPs to ensure that it is not degraded by intrinsic nucleases in the host. Compared with other the vaccination strategies, an mRNA-LNP vaccine can simulate host mRNA *in vivo*, which can enhance its stability and translation efficiency. Moreover, efficient manufacturing capabilities ensure that clinical batches of an mRNA vaccine can be generated within weeks after the sequence encoding the immunogen is obtained (45).

Roth et al. developed an mRNA-LNP vaccine, called DENV1-NS, which could prompt an intense T cell response. CD8 T cells prioritize NS3, NS4B, and NS5 as targets (46); therefore, the immunogenic region of DENV1-NS is formed by NS3, NS4B, and NS5 of the virus. The DENV1-NS mRNA was injected at 10 or 2 g, followed by a 28-day booster vaccination. Experiments showed that humanized HLA transgenic mice mounted a robust antiviral CD8+ T cell response, which reduced the infection of DENV1. Further experiments are necessary to demonstrate whether DENV1-NS can induce protection against other DENV serotypes (47).

Recently, Wollner et al. proposed the construction of a lipid nanoparticle-encapsulated mRNA vaccine against DENV serotype 1 that encoded membrane and envelope structural proteins (prM/E mRNA-LNP), with nucleotide modifications (48). The vaccine induced neutralizing antibody responses at both high (10 µg) and low (3 µg) doses through a primary booster strategy of intramuscular injection, which induced high EC50 (half maximal effective concentration) titers for serum neutralization, reaching 1/400. Moreover, CD4+ and CD8+ T cells with anti-DENV1 activity were induced after vaccination. Additionally, comparing the mice infected with DENV1 to the mice vaccinated with prM/E mRNA-LNP, they found that antibody-dependent enhancement (ADE) levels were 8-fold higher in the latter group (48).

#### 3.1.4 Protein vaccine candidates

Protein vaccines compare favorably to LATV in terms of safety profiles, suitability for immunocompromised individuals, and relative ease in obtaining tetravalent formulations (49). Hitherto, V180, developed by Merck Sharpe & Dohme (MSD), is one of the best prospective protein vaccines, which comprises a truncated form of the protein DEN-80E expressed in insect cells (22). Preclinical testing of the DEN-80E proteins from the four DENV serotypes with diverse adjuvants, including aluminum hydroxide Alhydrogel™ (Brenntag Biosector, Frederikssund, Denmark) and the investigational saponin-based ISCOMATRIX™ adjuvant (CSL Behring, King of Prussia, PA, USA), demonstrated their immunogenicity and efficacy (50). V180 induced excellent neutralizing antibody titers when combined with the ISCOMATRITM adjuvant. In addition, the group comprising a low-dose with 60 ISCOTM units produced the highest antibody titers targeting the four serotypes DENV. The antibody levels of the group with the ISCOMATRITM adjuvant declined over time, but remained at baseline levels even after one year, while the other groups treated with aluminum adjuvant and unadjuvanted formulations dropped to baseline levels after six months (51). However, the trial found that all V180 formulations were well tolerated, except that use of the ISCOMATRIXTM adjuvant correlated with an increased incidence of injection-site adverse events, as well as systemic adverse events and fever. Furthermore, this trial showed that higher and longer-lasting antibody levels were obtained using a series of three injections at an interval of 1 month. Nevertheless, further research into protein-based vaccines for DENV is necessary (51).

#### 3.1.5 DNA vaccine candidates

A DNA vaccine comprises a plasmid containing one or more genes encoding specific antigens. There has been progress in developing a tetravalent DNA vaccine (TVDV) from the US Naval Medical Reach Center (NMRC) and the WRAIR using the nucleic acid immunization method. In the TVDV, four monovalent double-stranded DNA vaccines are included in equal quantities. Using the plasmid VR1012 as the backbone, the monovalent plasmids carry the prM and E genes from DENV 1, 2, 3, or 4 (52). TVDV together with the cationic lipid-based adjuvant Vaxfectin were administered to three groups of volunteers in a phase I clinical trial at 0, 30, and 90 days. The results revealed that although TVDV appeared to be well tolerated, antibodies against only one, three, or all four dengue virus serotypes were detected in five out of 17 subjects between days 120 and 270 of the study. And another participant was found show an immune response on day 180 (52). The stability, low cost, and easy preparation of DNA vaccines are advantages compared with other vaccine candidates. Therefore, strengthening their immunogenicity is an urgent requirement.

### 3.2 Zika virus vaccine candidates

Associated with a high infection rate and a broader geographical distribution, Zika virus belongs to the Flaviviridae family (53). It is a positive-sense single-stranded RNA virus with two noncoding segments (5′ and 3′ respectively) around a single open reading frame (54). The open reading frame expresses a polyprotein that is cleaved into the capsid, premembrane, envelope, and seven nonstructural proteins (54). The transmission of the Zika virus mainly involves Aedes mosquitoes (55) and maternal transmission (56). Zika virus infection has complicated symptoms, including acute fever (57), neurological complications (58), and adverse fetal outcomes (53). The infection rate of the Zika virus has increased in the recent ten years; therefore, scientists are devoted to determining its pathogenic mechanism, which will lead to the development of an effective vaccine and accurate diagnostic methods. Without a vaccine or specific treatment for the Zika virus, the only available solution is vector control, has not proven to be satisfactory (19).

**Figure 4:**
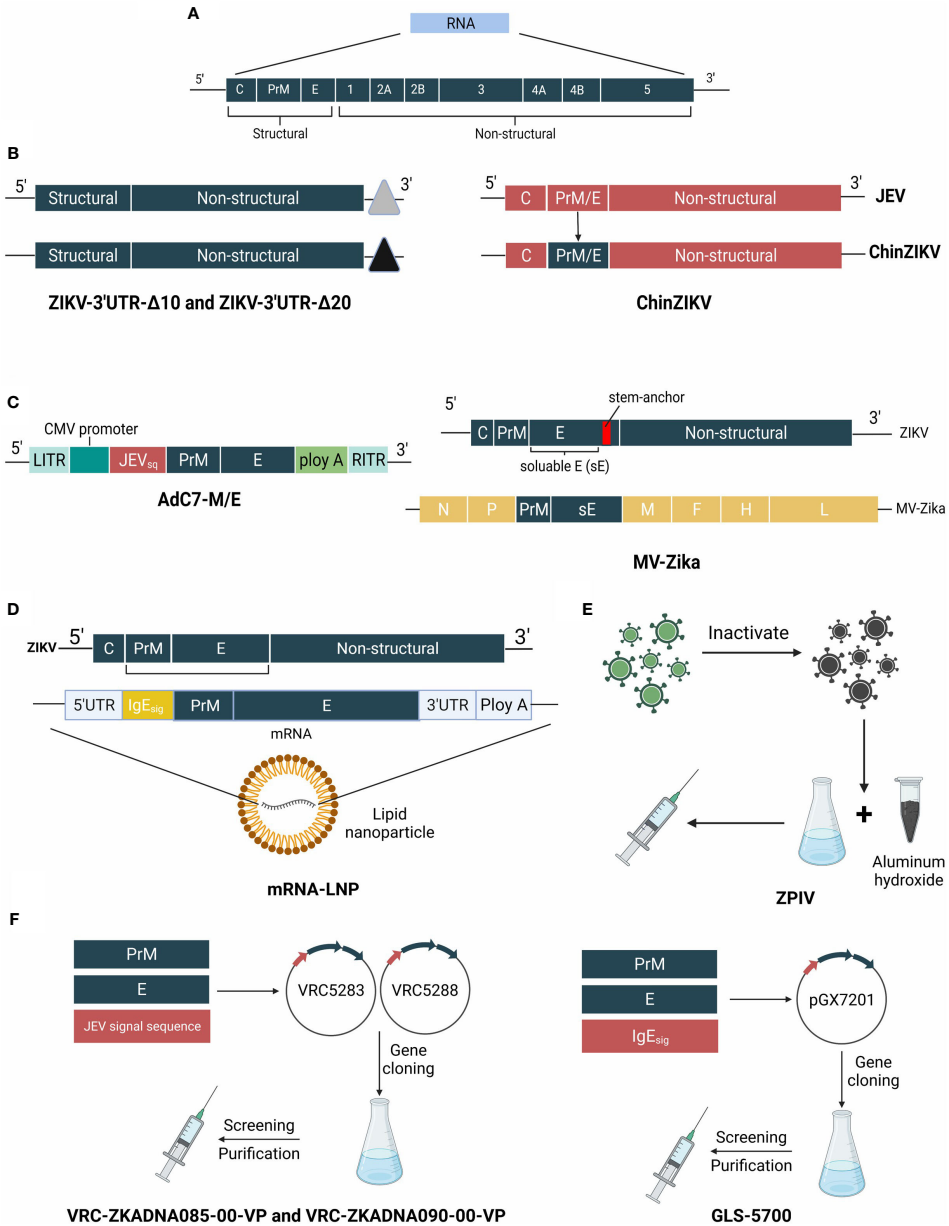
The genome of ZIKV and structure schematic diagrams of ZIKV vaccine candidates. **(A)** The genome of ZIKV is a positive-sense single-stranded RNA virus that encodes the capsid, precursor of membrane, envelope, and 7 nonstructural proteins. **(B)** ZIKV-3′UTR-Δ10 and ZIKV-3′UTR-Δ20 possess a 10-nucleotide or 20-nucleotide deletion within the 3′ UTR. ChinDENV used the Japanese encephalitis live-attenuated vaccine SA14-14-2 as the backbone, and expresses prM/E proteins. **(C)** MV-Zika takes live attenuated measles virus as a viral vector and expresses prM and soluble **(E)** AdC7-M/E uses recombinant chimpanzee adenoviruses as the backbone, and expresses the prM/E genes of ZIKV. **(D)** mRNA- LNP of ZIKV vaccine is encapsulated in lipid nanoparticles and encodes prM/E genes. **(E)** ZPIV is a purified formalin-inactivated ZIKV vaccine from strain PRVABC59. **(F)** VRC-ZKADNA085-00-VP (VRC-ZKADNA090-00-VP) creates VRC5283 (VRC5288) by replacing the ZIKV prM signal sequence with the analogous region of the Japanese encephalitis virus. GLS-5700 constructs pGX7201 to express prM/E proteins.

Developing a reliable and efficient vaccine to mitigate the ZIKV outbreak is urgently needed because of its deleterious impact on humans. Structural diagrams of ZIKV and its candidate vaccines are shown in **Figure 4**.

#### 3.2.1 Inactivated vaccine candidates

Abbink et al. developed a purified formalin-inactivated ZIKV vaccine. As part of phase I clinical trials, Abbink et al. assessed its protective efficacy and adoptive transfer. Based on the results of enzyme-linked immunosorbent assays (ELISAs) and 50% neutralization titer (MN50) assays, the median log antibody titers increased substantially to 3.54 and 3.66 with two methods respectively. Naive Balb/c mice infused with two higher doses of pure IgG from ZIKV PIV-vaccinated monkey plasma at week 8 became fully protected from ZIKV PIV infection. Three institutes (The Saint Louis University (SLU), The WRAIR, and The Beth Israel Deaconess Medical Center (BIDMC)) carried out the clinical trials for ZPIV (inactivated vaccine). The report showed that participants at the BIDMC generated the highest GMTs compared with the two other groups. Although the GMTs of the three groups declined a little from day 43 to day 57, they remained higher than the baseline titers (59, 60).

#### 3.2.2 DNA vaccine candidates

A novel DNA vaccine, named GLS-5700, a synthetic, consensus-coding DNA vaccine, was created by Tebas et al., which contains the premembrane and envelope gene regions of ZIKV. In the study, 40 participants were divided into two groups, and each group received either 1 mg or 2 mg of vaccine at baseline, 4 weeks, and 12 weeks. The results revealed that at two weeks after the third dose, all participants produced antibodies specific to ZIKV. GLS-5700 induced neutralizing antibody titers of 1642 in the 1-mg dose group and 2871 in the 2-mg group. Notably, serum from five participants with unmeasured neutralizing titers produced protective effects in A129 mice, which showed that the vaccine generated protective efficacy (61). However, this did not correlate with the vaccination dose in 60% of participants after vaccination, and their neutralizing titers ranged between 1:18 and 1:317 (62).

Dowd et al. developed other DNA vaccines: VRC-ZKADNA085-00-VP (VRC5283) and VRC-ZKADNA090-00-VP (VRC5288) (63). In VRC5283, an analogous region of the Japanese encephalitis virus (JEV) was substituted for the ZIKV prM signal sequence. Similarly, the chimeric ZIKV/JEV prM-E construct, VRC5288, comprised the last 98 amino acids of E, the stem, and transmembrane regions (ST/TM), which enhanced SVP secretion (64). VRC-ZKADNA085-00-VP was found to produce the highest neutralizing antibody titers (geometric mean titer 120) with vaccination at weeks 0, 4, and 20. VRC ZKADNA090-00-VP generated the highest GMT of 304 *via* a needle-free injection device and a split dose of two 5 ml injections at weeks 0, 4, and 8 (65). The results revealed that they were both safe and well tolerated. Neutralizing titers were observed in 100% of participants (63).

#### 3.2.3 mRNA vaccine candidates

Medina-Magües et al. reported an mRNA vaccine encoding the pre-membrane and envelope (prM/E) glycoproteins of ZIKV strain Brazil SPH2015, which was encapsulated in LNPs, named mRNA-LNP (66). Compared with other vaccine candidates, mRNA vaccines avoid infection and insertional mutagenesis risks, as well as anti-vector immunity. Besides, mRNA vaccines are more efficiently delivered, taken up, and expressed by their target cells (67). Using the AG129 mice and BALB/c mice models, the authors demonstrated that vaccination with mRNA-LNP induced host-protective antibodies in immunized animals without causing any post-vaccination side effects (66). Using immunocompetent rhesus macaques, Pardi et al. demonstrated that a single intradermal injection of mRNA-LNP modified by nucleosides generated potent neutralizing antibodies and specific antibodies against ZIKV, which provided complete protection against virus challenge (68). Additionally, Richner et al. developed an mRNA-1325 vaccine expressing prM-E, which protected mice against lethal ZIKV challenge and conferred sterilization immunity (69).

#### 3.2.4 Viral vector vaccine candidates

Using rVSV, Betancourt et al. independently expressed two versions of ZIKV: One that contained the precursor to the membrane protein (ZprME) and one that did not (70). They found that mice injected with the vaccine produced Ig against ZIKV, meaning that it could neutralize ZIKV infection *in vitro*. According to Nürnberger et al, mice could be protected against Zika infection by MV-Zika-sE, which comprised an attenuated measles virus vector-based vaccine expressing the prM and soluble E proteins (71). Besides, Xu et al. developed a ZIKV vaccine comprising recombinant AdC7 expressing the ZIKV M/E proteins (AdC7-M/E) (72). Exploiting the advantages of recombinant chimpanzee adenoviruses, such as their safety profile, adjuvanticity, and low level of preexisting immunity in humans (73), recombinant AdC7 has been used as viral vector for vaccines against influenza virus, HIV, and respiratory syncytial virus (RSV) (74–76). AdC7-M/E was not only highly immunogenic, but also achieved sterilizing immunity. These results demonstrated that AdC7-M/E might be a promising vaccine candidate against ZIKV.

#### 3.2.5 Live attenuated vaccine candidates

There are two strategies to develop a live attenuated vaccine: (i) Mutating a real ZIKV isolate to produce attenuating mutations and (ii) creating chimeric flaviviruses containing the ZIKV prM-E gene using JEV or YFV (77). Shan et al. developed two candidates for live attenuated ZIKV vaccines, which contain 10-nucleotide segment (ZIKV-3′UTR-10) or 20-nucleotide (ZIKV-3′UTR-Δ20) deletions in the 3′ UTR of the ZIKV genome from a pre-epidemic Cambodian strain FSS13025 (78). After single dose vaccination, the 20-nucleotide deletion ZIKV vaccine candidate led to neutralizing antibody titers greater than 1:1,000 and provided sterilizing immunity in monkeys. Importantly, both vaccine candidates showed good safety profiles (79). Similar to ChinDENV, Li et al. developed a recombinant chimeric ZIKV vaccine (ChinZIKV) that expressed the prM and E proteins of ZIKV, adopting the licensed JEV attenuated vaccine SA14-14-2 as the backbone (80). Single dose vaccination with ChinZIKV produced strong and durable immune responses and totally protection from ZIKV challenge.

### 3.3 CHIKV vaccine candidates

**Figure 5:**
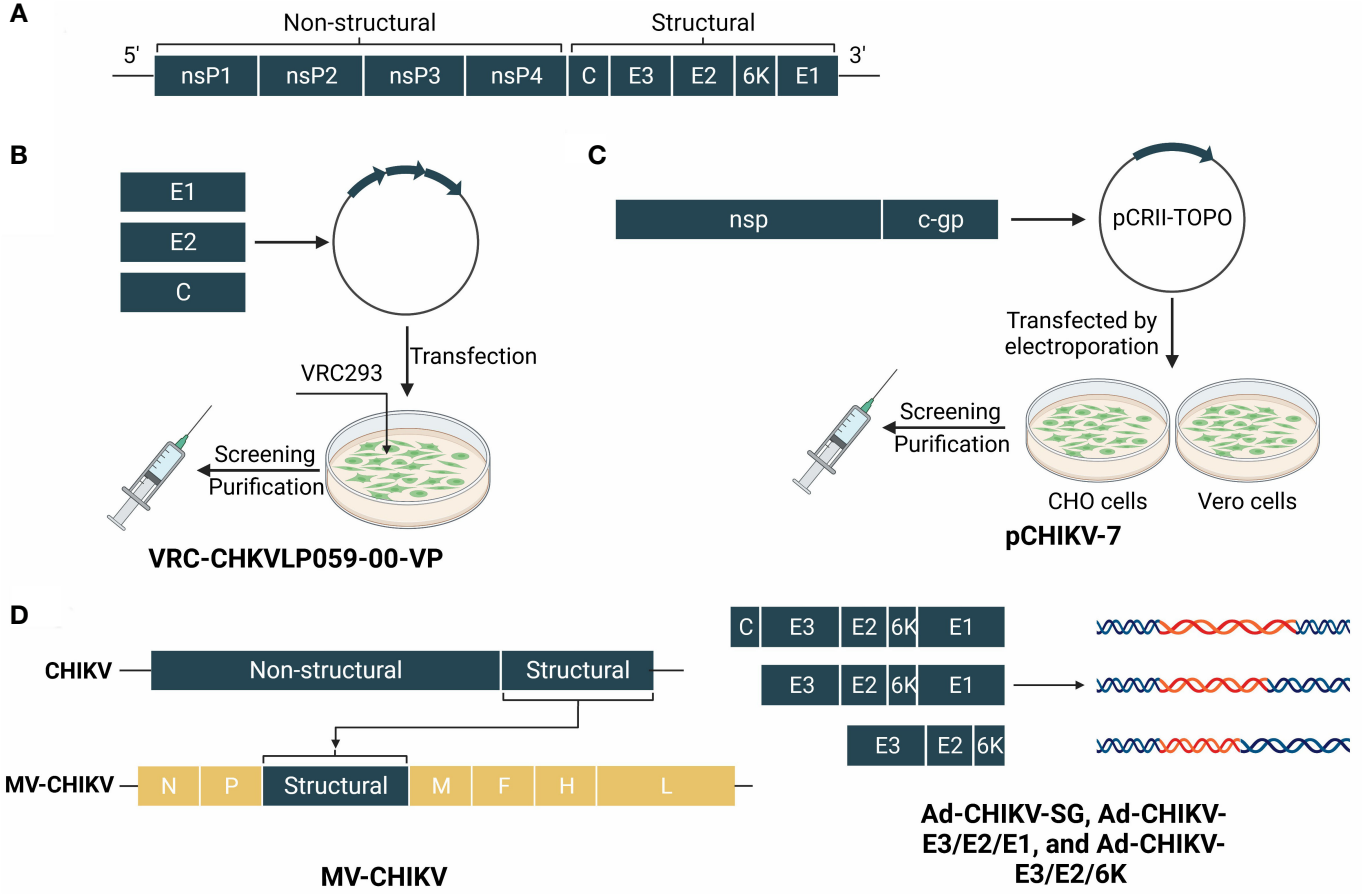
The genome of CHIKV and structure schematic diagrams of CHIKV vaccine candidates. **(A)** The genome of CHIKV encodes a capsid and a phospholipid envelope, and comprises a single-stranded RNA genome. The polyproteins are cropped into four non-structural proteins (nsP1–4) and five structural proteins. **(B)** VRC-CHKVLP059-00-VP is produced by human embryonic kidney VRC293 cells from a DNA plasmid encoding the structural genes of the chikungunya virus. **(C)** pCHIKV-7 is an iDNA vaccine that encodes the full-length infectious genome of live attenuated CHIKV clone 181/25 downstream from a eukaryotic promoter. **(D)** MV-CHIK takes the measles virus as a viral vector and inserts structural genes of CHIKV. Ad-CHIKV-SG, Ad-CHIKV-E3/E2/E1, and Ad-CHIKV-E3/E2/6K insert three groups of different structural proteins into the adenovirus.

The Chikungunya virus (CHIKV) is part of the Semliki Forest antigenic group of the Alphaviridae family, which also contains other alphaviruses known to cause arthritic conditions (81). CHIKV consists of three major components: a capsid, an envelope, and a single stranded RNA genome. In terms of genotypes, it can be divided into four categories: East-Central-South Africa, West Africa, Asian, and Indian Ocean lineages (19). The viral polyprotein comprises four nonstructural proteins (nsP1–4), which comprise the viral replication machinery, and five structural proteins (82). The transmission of CHIKV occurs *via* Aedes mosquitoes (83) and vertical propagation (84). Patients with CHIKV infection suffer from polyarthralgia and myalgia after an initial incubation period. The obvious feature of Chikungunya fever is a high viremic load and concomitant abnormalities, such as fever, rash, and severe joint and muscle pain. The incubation period of CHIKV infection has not been accurately determined (85). Structural diagrams of CHIKV and its candidate vaccines are shown in **Figure 5**.

#### 3.3.1 Virus-like particle vaccine candidates

Human cells can be transfected with DNA expression plasmids to produce VLPs containing the structural cassette for CHIKV. Although VLPs are similar to intact virions, they cannot replicate because of the absence of genomic viral RNA (86). Chang et al. developed VRC 311, a VLP CHIKV vaccine, which was tested in an open-label, incremental dose, phase 1 trial (87). Participants were divided into three groups receiving 10 μg, 20 μg, or 40 μg of the vaccine on weeks 0, 4, and 20. Follow-up for 44 weeks after admission demonstrated that all injections were well tolerated with good safety profiles. After the third vaccination, the GMTs of the half-maximum inhibitory concentrations increased to 8745, 4525, and 5390 for the three groups, respectively, which proved that the CHIKV VLP vaccine was immunogenic. Using a similar principle, Reisinger et al. developed a measles-vectored and live-attenuated CHIKV vaccine (MV-CHIK) (88) and proved that the vaccine had promising immunogenicity following two vaccinations and an acceptable safety and tolerability profile.

#### 3.3.2 Live attenuated vaccines candidates

Plante et al. reported a rational attenuation mechanism in which the subgenomic promoter in a cDNA CHIKV clone was replaced by the internal ribosome entry site (IRES) from encephalomyocarditis virus, which could prevent the infection of mosquito vectors and could not replicate in mosquito cells or infect mosquitoes *in vivo* (89). Additionally, the nonstructural CHIKV components might strengthen the immune response specific to CHIKVs and decrease the attenuation that frequently accompanies chimeric alphaviruses (90). According to Taylor et al., the CHIKV capsid protein has an uncharacterized nucleolar localization sequence (NoLS) in the N-terminal region, which might be exploited to develop vaccines (91). Using site-directed mutagenesis, Taylor et al. decreased the effectiveness of nuclear import of the CHIKV capsid protein. They found that mammalian and mosquito cells demonstrated reduced replication of the virus when this site was mutated (CHIKV-NoLS). Compared with mice infected with wild-type CHIKV, C57BL/6 mice infected with CHIKV-NoLS did not exhibit disease symptoms (91). Therefore, this approach could be considered as a method to develop live attenuated vaccines.

#### 3.3.3 Protein vaccine candidates

A CHIKV subunit vaccine was first developed in insect cells by Metz et al. (92), which were chosen for their ability to express E1 and E2 in different expression systems. Additionally, they also examined the immunogenicity of three CHIKV VLPs, and recombinantly produced E1 and E2 baculoviruses. The results revealed that the effectiveness of the subunit vaccines was lower than that of the CHIKV VLPs and other vaccine candidates (93). Hence, more research has been carried out to enhance the efficacy of subunit vaccines. Kumar et al. demonstrated that with the use of adjuvants (including alum, Mw, CadB (rE2p), alum/Mw (formalin inactivated CHIKV), and alum (BPL-inactivated CHIKV)), the overall efficacy of the subunit vaccines was strengthened, with alum being was the most effective adjuvant (94).

#### 3.3.4 DNA vaccine candidates

Taking advantage of the advantages of DNA vaccines, Hidajat et al. developed a new approach called infectious clone technology (iDNA^®^), which could produce vaccines comprising plasmid DNA *in vitro* and *in vivo* (95). This advanced technology enabled them to generate the full-length cDNA of the 181/25 vaccine for CHIKV (pCHIKV-7) (96). *In vivo*, neutralizing antibodies were detected in all experimental animals and a single dose vaccination could protect the mice from CHIKV challenge. Muthumani et al. demonstrated the advantages of vaccination therapy using a combination of active and passive antibody therapies. They used electroporation-mediated delivery to generate synthetic DNA plasmids encoding anti-CHIKV envelope monoclonal antibodies (dMAbs) that were biologically active (97). The results revealed that the speed of antibody production *in vivo* was faster than with active anti-CHIKV DNA vaccination after one intramuscular injection of dMAbs, and protected the mice from CHIKV challenge.

#### 3.3.5 Recombinant virus-vectored vaccine candidates

In many vaccine development programs, genes encoding protective antigens are inserted into the virus genome vector to generate recombinant virus-vectored vaccines. Recently, Rossi et al. reported the development of a new measles virus-vectored vaccine against CHIKV (MV-CHIK) (98). In a phase II clinical trial, all participants were shown to produce neutralizing antibodies after one or two immunizations, with GMTs ranging from 12.87 to 174.80 and seroconversion rates ranging from 50.0% to 95.9%. In addition, no serious adverse events have been reported as a result of the clinical trial. Apart from that, the clinical trials explored the influence of vaccine dose, vaccination time, and booster dose. A booster dose at 1 month or 6 months significantly enhanced the neutralizing antibody titers. More importantly, the booster dose at 6 months showed higher neutralizing antibody titers than the booster dose at 1 month, prompting us to think of a better approach to the vaccination and booster dose (88). Therefore, the excellent safety profile, tolerability, and immunogenicity of MV-CHIK suggests it as a promising choice for vaccination. Dora et al. reported an oral, rather than injectable, CHIKV vaccine based on adenovirus (99). Using different CHIKV structural protein combinations, they developed three vaccine candidates: Ad-CHIKV-SG, Ad-CHIKV-E3/E2/E1, and Ad-CHIKV-E3/E2/6K. The results revealed that a single administration could induce high titers of antibodies in BALB/c and C57BL/6 strains of mice (99).

### 3.4 Comparison of vaccine candidates

The live attenuated vaccine candidates and inactivated vaccine candidates, as traditional vaccine platforms, have contributed significantly to the development of many viral vaccines because of their proven vaccine development systems and the advantage of producing excellent neutralizing antibody titers. Currently, live attenuated and inactivated vaccine candidates are the main vaccines in clinical trials. However, most of these vaccines, such as CYD-TDV and TAK-003, are not suitable for certain groups of people, including infants and the elderly. Furthermore, because various vaccines use different virus backbones, they will generate different neutralizing antibodies against different serotypes. Protein vaccine candidates possess a good level of safety and activate the host’s immune system to combat the virus (100). However, protein vaccines also need proper adjuvants and vaccine formulations to generate high protective efficacy. For V180 (a protein vaccine for DENV), even improving the dose of vaccine or adjuvants (high-dose vaccine and high ISCO™ units) did not induce the highest neutralizing antibody titers. As a new platform for vaccine development, nucleic acid-vaccines are now being utilized to treat a variety of infectious diseases and some cancers. Notably, since nucleic acid-vaccine candidates can be flexibly designed to target different pathogens according to their goal targets, it enables the design and production of vaccines in a short period, which provides opportunities for industrial production. After vaccination, nucleic acid-vaccine candidates can mimic viral infections *via in situ* expression of vaccine antigens, which will induce humoral and cellular immunity (101). However, as mentioned above, TVDV (a DNA vaccine candidate for DENV) only induced a low level of neutralizing antibodies in two participants. DNA vaccines can induce lower neutralizing antibody titers than the live attenuated vaccine candidates or inactivated vaccine candidates, which means that DNA vaccines always need an adjuvant to enhance the immune response or an efficient delivery system. To date, no mRNA vaccine for these three diseases has entered clinical trials. mRNA vaccines do not produce infectious particles or transform the host cells’ genomes into their own. They can also complete the whole vaccine design with mature manufacturing processes. As mentioned above, the University of Pennsylvania and Moderna Therapeutics developed an mRNA vaccine for the ZIKV. They both generated excellent protection in mice. Besides, given the mutation of virus proteins, mRNA vaccine sequences can be designed to target mutation sites. Generally, an mRNA vaccine is a potential vaccine development platform for Mosquito-borne diseases because of its scalability, cheap manufacturing cost, and fast production. Viral-vector vaccines have an excellent track record of inducing immunity, and adenovirus-vectored vaccines are currently considered to be a high-potential high-tech vaccine platform (102). MV-CHIK (viral-vectored vaccine for CHIKV) showed different vaccine protective efficacies at different times of vaccination and booster doses. The difference in duration between two vaccination and booster doses can be as long as six months. Hence, it is hypothesized that different inoculation routes might produce different immune responses. Virus-like particles (VLP) vaccine maintain the virus particle structure and display multimeric antigens (103). In contrast to inactivated and live attenuated vaccine candidates, VLP vaccines lack viral genomes and possess a good safety profile, which might make them more suitable for elderly people and immunocompromised individuals. VRC-CHKVLP059-00-VP (a VLP vaccine for CHIKV) induced the highest neutralizing antibody titers (8745) at week 24. Therefore, the VLP vaccine, as a preventive vaccine, is a promising vaccine platform against Mosquito-borne diseases. Besides, a VLP vaccine can present patients’ antigens and help them fight against chronic and metabolic diseases or virus-associated cancers (104).

## 4 Challenges in vaccine development

Immunopathological events play a crucial role in the development of vaccines for DENV and ZIKV. These viruses can infect immune cells such as monocytes and dendritic cells, triggering inflammation and the production of antiviral cytokines. However, uncontrolled inflammation can lead to exacerbation and immune response validation in the context of DENV infection. Studies by Patro et al. have shown that DENV infection generates an inflammatory response, leading to increased concentrations of pro-inflammatory cytokines. Such an excessive immune response can cause vascular leakage (105) and hemorrhage (106). Therefore, it is important to consider the immunopathology of these viruses while developing vaccines to mitigate potential adverse events and hemorrhage. Studies have demonstrated that the immune response to the envelope protein and non-structural protein NS1 of the flavivirus genus can cause cross-reactivity among different serotypes of dengue virus (107, 108). This cross-reactivity may contribute to the development of hemorrhagic symptoms. Therefore, it is crucial to monitor changes in cytokines and chemokines caused by the vaccine during its development to prevent adverse immune events from occurring.

Immune responses to DENV are primarily based on antibodies directed against the prM and fusion loop epitopes (FLE) of the virus, which will produce cross-reactions between different DENV serotypes and might promote antibody-dependent enhancement (ADE) (109). The anti-FLE antibodies will promote Fcγ receptor-mediated entry into the host, which enhances the replication of other serotypes. PrM is predominantly found in immature viral particles. Although the host generates anti-prM antibodies during viral infection and they promote Fc uptake, these antibodies do not possess a potent neutralizing ability against the virus (110). On the contrary, the approach currently used for developing dengue virus (DENV) and Zika virus (ZIKV) vaccines may actually increase the likelihood of antibody-dependent enhancement (ADE) of infection. This underscores the need to explore alternative strategies for reducing ADE and improving the vaccines’ ability to stimulate a specific immune response. One promising approach is to design the vaccines to target potent neutralizing epitopes, such as the ED3 and EDE regions. Robinson et al. demonstrated the effectiveness of this approach by showing that an anti-ED3 monoclonal antibody provided robust protection in humanized mice infected with dengue virus (111). The research should focus on identifying epitopes that may trigger Antibody-Dependent Enhancement (ADE) phenomena, such as prM and FLE mentioned previously. In addition, the interaction between DENV and ZIKV poses a challenge to vaccine research. Therefore, it is crucial to consider these factors in developing effective vaccines against these viruses (112). Research demonstrated that ZIKV could strengthen the future risk of Dengue infection (113). Anti-FLE antibodies produced by DENV infection are able to bind to Fcγ and mediate the entry of ZIKV virus into the host cell, enhancing the symptoms produced by ZIKV infection. Hence, it is worth considering how to diminish the influence of DENV and ZIKV. Furthermore, considering the four different DENV serotypes, sequential and multi-dose vaccinations are essential to provide sufficient protective capacity.

Currently these is no licensed vaccine against CHIKV. More importantly, no specific antiviral is available to control CHIKV replication; therefore, supportive and symptomatic measures are needed (114). It is a huge problem that we do not know all the CHIKV serotypes (115), which will lead to less effective CHIKV vaccines. At the meanwhile, we still need to pay attention to the ADE phenomenon, although there are no definite reports of CHIKV ADE, but there have been findings of ADE phenomenon *in vitro* with the closely related alphavirus RRV (116). How to establish a balance between immunogenicity and the safety profile is also a difficult challenge, which is related with the health of test subjects.

Besides the previously mentioned considerations in vaccine design, ensuring the safety of the vaccine is a crucial aspect. When developing vaccines for DENV and ZIKV, it is essential to produce long-lasting and stable protective antibodies against each serotype of the virus. This will prevent infections from different serotypes, which can cause severe immune reactions. Some research indicates that secondary DENV infections result in more severe symptoms, possibly due to the initial heterozygous DENV infection (117). As a result, ongoing vaccine research for these three viruses is taking measures to prevent the occurrence of such events.

In theory, the vaccine should not be developed to produce serious adverse reactions in naive or previously infected people. However, in the current study, Dengvaxia^®^, the most successful vaccine to date, caused serious adverse reactions in trials with natural vaccinees (118). In contrast, clinical trials have shown that TAK-003 has much better safety outcomes than Dengvaxia^®^.

## 5 Conclusions

Despite decades of progress, Mosquito-borne diseases remain a challenge for mankind. A Solidarity Vaccine Trial has been proposed by the WHO; however, some vaccine manufacturers and researchers might take extreme measures to determine which vaccine is most effective, which is irresponsible (119). No country can evade these public health issues. Compared with insect vector control, useful vaccines and medications are the most therapies powerful to stop the spread of infectious diseases.

## Author contributions

ZWH and YXZ wrote the manuscript. JJZ helped to analyze the data. WCS helped to prepare the figures. KDC was responsible for conceptualization and data curation. HYL, YJZ and YLL were involved with review ang editing. All authors contributed to the article andapproved the submitted version.
