# Supplementary material for: Vaccine development for mosquito-borne viral diseases
Source: Front Immunol. 2023 May 12;14:1161149. doi: 10.3389/fimmu.2023.1161149 (PMC10213220; doi:10.3389/fimmu.2023.1161149)
Supplement: Supplementary file 1 [file DataSheet_1.pdf]

References cited in the Supplementary Material:

1. Sabchareon A, Wallace D, Sirivichayakul C, Limkittikul K, Chanthavanich P, Suvannadabba S, et al. Protective efficacy of the recombinant, live-attenuated, CYD tetravalent dengue vaccine in Thai schoolchildren: a randomised, controlled phase 2b trial. Lancet. 2012;380(9853):1559-67.
2. Kumar M, Sudeep AB, Arankalle VA. Evaluation of recombinant E2 protein-based and whole-virus inactivated candidate vaccines against chikungunya virus. Vaccine. 2012;30(43):6142-9.
3. Hidajat R, Nickols B, Forrester N, Tretyakova I, Weaver S, Pushko P. Next generation sequencing of DNA-launched Chikungunya vaccine virus. Virology. 2016;490:83-90.
4. Kallas EG, Precioso AR, Palacios R, Thome B, Braga PE, Vanni T, et al. Safety and immunogenicity of the tetravalent, live-attenuated dengue vaccine Butantan-DV in adults in Brazil: a two-step, double-blind, randomised placebo-controlled phase 2 trial. Lancet Infect Dis. 2020;20(7):839-50.
5. Li Z, Yang H, Yang J, Lin H, Wang W, Liu L, et al. Construction and preliminary investigation of a novel dengue serotype 4 chimeric virus using Japanese encephalitis vaccine strain SA14-14-2 as the backbone. Virus Res. 2014;191:10-20.
6. Diaz C, Koren M, Lin L, Martinez LJ, Eckels KH, Campos M, et al. Safety and Immunogenicity of Different Formulations of a Tetravalent Dengue Purified Inactivated Vaccine in Healthy Adults from Puerto Rico: Final Results after 3 Years of Follow-Up from a Randomized, Placebo-Controlled Phase I Study. Am J Trop Med Hyg. 2020;102(5):951-4.
7. Swaminathan G, Thoryk EA, Cox KS, Meschino S, Dubey SA, Vora KA, et al. A novel lipid nanoparticle adjuvant significantly enhances B cell and T cell responses to sub-unit vaccine antigens. Vaccine. 2016;34(1):110-9.
8. Roth C, Cantaert T, Colas C, Prot M, Casademont I, Levillayer L, et al. A Modified mRNA Vaccine Targeting Immunodominant NS Epitopes Protects Against Dengue Virus Infection in HLA Class I Transgenic Mice. Front Immunol. 2019;10:1424.
9. Wollner CJ, Richner M, Hassert MA, Pinto AK, Brien JD, Richner JM. A Dengue Virus Serotype 1 mRNA-LNP Vaccine Elicits Protective Immune Responses. J Virol. 2021;95(12).
10. Musso D, Nilles EJ, Cao-Lormeau VM. Rapid spread of emerging Zika virus in the Pacific area. Clin Microbiol Infect. 2014;20(10):O595-6.
11. Meaney-Delman D, Hills SL, Williams C, Galang RR, Iyengar P, Hennenfent AK, et al. Zika Virus Infection Among US Pregnant Travelers - August 2015-February 2016. Mmwr-Morbid Mortal W. 2016;65(8):211-4.
12. Xu K, Song Y, Dai L, Zhang Y, Lu X, Xie Y, et al. Recombinant Chimpanzee Adenovirus Vaccine AdC7-M/E Protects against Zika Virus Infection and Testis Damage. J Virol. 2018;92(6).
13. Sharma A, Wendland R, Sung B, Wu W, Grunwald T, Worgall S. Maternal immunization with chimpanzee adenovirus expressing RSV fusion protein protects against neonatal RSV pulmonary infection. Vaccine. 2014;32(43):5761-8.
14. Dora EG, Rossi SL, Weaver SC, Tucker SN, Mateo R. An adjuvanted adenovirus 5-based vaccine elicits neutralizing antibodies and protects mice against chikungunya virus-induced footpad swelling. Vaccine. 2019;37(24):3146-50.
15. Rossi SL, Comer JE, Wang E, Azar SR, Lawrence WS, Plante JA, et al. Immunogenicity and Efficacy of a Measles Virus-Vectored Chikungunya Vaccine in Nonhuman Primates. J Infect Dis. 2019;220(5):735-42.
16. Modjarrad K, Lin L, George SL, Stephenson KE, Eckels KH, De La Barrera RA, et al. Preliminary

aggregate safety and immunogenicity results from three trials of a purified inactivated Zika virus vaccine candidate: phase 1, randomised, double-blind, placebo-controlled clinical trials. Lancet. 2018;391(10120):563-71.
17. Dowd KA, Ko SY, Morabito KM, Yang ES, Pelc RS, DeMaso CR, et al. Rapid development of a DNA vaccine for Zika virus. Science. 2016;354(6309):237-40.
18. Gaudinski MR, Houser KV, Morabito KM, Hu Z, Yamshchikov G, Rothwell RS, et al. Safety, tolerability, and immunogenicity of two Zika virus DNA vaccine candidates in healthy adults: randomised, open-label, phase 1 clinical trials. Lancet. 2018;391(10120):552-62.
19. Medina-Magues LG, Gergen J, Jasny E, Petsch B, Lopera-Madrid J, Medina-Magues ES, et al. mRNA Vaccine Protects against Zika Virus. Vaccines (Basel). 2021;9(12).
20. Vu DM, Jungkind D, Angelle Desiree L. Chikungunya Virus. Clin Lab Med. 2017;37(2):371-82.
21. Powers AM. Vaccine and Therapeutic Options To Control Chikungunya Virus. Clin Microbiol Rev. 2018;31(1).
22. Taylor A, Liu X, Zaid A, Goh LY, Hobson-Peters J, Hall RA, et al. Mutation of the N-Terminal Region of Chikungunya Virus Capsid Protein: Implications for Vaccine Design. mBio. 2017;8(1).
23. Khan AH, Morita K, Parquet MDC, Hasebe F, Mathenge EGM, Igarashi A. Complete nucleotide sequence of chikungunya virus and evidence for an internal polyadenylation site. J Gen Virol. 2002;83(Pt 12):3075-84.
24. Metz SW, Martina BE, van den Doel P, Geertsema C, Osterhaus AD, Vlak JM, et al. Chikungunya virus-like particles are more immunogenic in a lethal AG129 mouse model compared to glycoprotein E1 or E2 subunits. Vaccine. 2013;31(51):6092-6.
